# Investigation of the expression levels of *CDH1*, *FHIT*, *PTEN*, and *TTPAL* genes in colorectal tumors

**DOI:** 10.3906/sag-2110-296

**Published:** 2021-12-12

**Authors:** Evrim Suna ARIKAN SÖYLEMEZ, Zafer SÖYLEMEZ, Murat ÇİLEKAR, Yüksel ARIKAN, Çiğdem TOKYOL, İbrahim Halil KENGER, Mustafa SOLAK

**Affiliations:** 1 Department of Medical Biology, Faculty of Medicine, Afyonkarahisar Health Sciences University, Afyonkarahisar, Turkey; 2 Department of General Surgery, Faculty of Medicine, Afyonkarahisar Health Sciences University, Afyonkarahisar, Turkey; 3 General Surgery Department, Park Hayat Hospital, Afyonkarahisar, Turkey; 4 Department of Pathology, Faculty of Medicine, Afyonkarahisar Health Sciences University, Afyonkarahisar, Turkey; 5 Department of Medical Genetics, Faculty of Medicine, Gaziantep Islam, Science and Technology University, Gaziantep, Turkey; 6 Department of Medical Genetics, Faculty of Medicine, Biruni University, İstanbul, Turkey

**Keywords:** *CDH1*, *FHIT*, *PTEN*, *TTPAL*, colorectal tumor, gene expression

## Abstract

**Background/aim:**

The main aim of the study is to assess expression levels of *CDH1*, *FHIT*, *PTEN*, and *TTPAL* genes in tumors and peripheral bloods of colorectal cancer patients in staged I–IV.

**Materials and methods:**

Gene expression analysis of related genes were performed for tumor tissues and peripheral blood samples of 51 colorectal cancer patients and colon tissues and blood samples of 5 healthy individuals. The real-time-PCR reaction method was used for the analysis.

**Results:**

Alteration of mRNA levels of related genes in tumor tissues of colorectal cancer cases was determined compared to control tissues. *GAPDH* and *TBP* were used for the normalization. While the mRNA levels of *CDH1* decreased, the mRNA level of the *FHIT* and *TTPAL* genes increased in the tumor tissues. There was no *PTEN* gene expression difference in tumor tissues (total). The mRNA levels of the *CDH1* and *PTEN* genes were increased while the mRNA levels of *FHIT* and *TTPAL* genes decreased in the blood (total). The mRNA levels of the *CDH1 g*ene decreased at each stage (I–IV) in the tumor tissues and increased at each stage (I–IV) in the blood. The *PTEN* gene mRNA levels at each stage were controversial. The mRNA levels of the *FHIT g*ene increased at stage I-II-III, decreased at stage IV in the tissues and decreased at each stage (I–IV) in the blood. The mRNA levels of *TTPAL g*ene increased at each stage (I–IV) in the tissues and decreased at each stage (I–IV) in the blood.

**Conclusion:**

Although related expression levels in tissue did not correlate with its expression in blood, consistent with previous studies *FHIT* and *TTPAL* genes upregulation and *CDH1* downregulation, in especially tumoral tissues, may serve as predictive determinants for the patients with colorectal cancer.

## 1. Introduction

Colorectal cancer (CRC) is the third most common cancer and one of the main causes of cancer-related deaths worldwide [1, 2]. The two main causes of CRC-related death result from the lack of early diagnosis and metastasis [3].

Etiologic and pathophysiologic factors of CRC are very wide. Epithelial-mesenchymal transition (EMT) is indispensable in the progression of these conditions [4]. The process of EMT is associated with decreased expression of epithelial markers such as E-cadherin (*CDH1*) [5]. The loss of E-cadherin during the EMT process leads to destroyed cell-cell adhesion, increased cell motility, and advanced stages of cancer [6]. E-cadherin is a member of a family of homophilic transmembrane glycoproteins expressed in almost all epithelial tissues and is responsible for calcium (Ca^+2^) dependent cell-cell adhesion. It also plays essential roles in establishing and maintaining cell polarity, cell signaling, cellular differentiation, and normal tissue morphology [7, 8, 9]. *CDH1* dysfunction has been reported in some of them due to allelic deletion and mutation [10, 11]. E-cadherin inactivation leads to activation of the β-catenin transcriptional activity [12]. Its constitutively activated form has been found in various human cancer types and up to 80% of tumors have a nuclear accumulation of β-catenin in colon cancer [13].

Abnormal transcripts of Fragile Histidine Triad (*FHIT*) have been found in approximately half of all esophageal, gastric and colon carcinomas and have been reported to be inactivated in many different types of cancer. The encoded protein is also a tumor suppressor because loss of its activity results in replication stress and DNA damage^1^. *FHIT* inactivation appears to be a later event, possibly associated with progression to more aggressive neoplasms [14].

Tumor suppressor *PTEN* (phosphatase and tensin homolog) has a leading role in a variety of processes associated with cell survival, proliferation, and growth [15]. In some tumors, the subcellular localization of PTEN protein seems to mediate its activity [16]. The absence of PTEN has been reported to be associated with more aggressive diseases and with high degree of neoplastic transformation, suggesting an important nuclear function for *PTEN* in tumor suppression [17, 18].

*TTPAL* (Tocopherol alpha transfer protein-like) is reported as a novel gene displayed hotspot mutations in the validation set samples [19]. *TTPAL* is possibly involved in the invasion and metastasis of CRC [20]. Besides, Gou et al. [21] reported that whole genome copy number profiling in primary colorectal tumor tissues has unravelled *TTPAL* as a top amplified gene CRC [21]. It is demonstrated that copy number gain of *TTPAL* leads to gene overexpression in CRC. It was also shown that *TTPAL* is an oncogene by promoting cell proliferation, migration and invasion in vitro and animal models. *TTPAL* was found to activate activated Wnt/β-catenin signalling, a key oncogenic pathway in CRC. They also suggested that *TTPAL* expression also serves as an independent prognostic marker for CRC patients [21].

In this study, expression level of *CDH1*, *FHIT*, *PTEN*, and *TTPAL* genes were analysed in CRC patients. In recent years, promising studies on molecular and biological characteristics of colorectal cancer have been reported to help understand cancer pathogenesis. Despite these studies, there is no definite opinion on genetic and genomic changes and their importance for colorectal tumorigenesis.

Recent studies on these changes will contribute to a better understanding of colorectal cancer pathophysiology. It can be concluded that with the early detection of colorectal cancer as a result of a long process under the influence of genetic and environmental factors, the patient will have more positive results in terms of diagnosis, prognosis and treatment. The importance of molecular biomarkers has emerged in early stage diagnosis.

## 2. Materials and methods

### 2.1. Human samples

This study is a continuation of our former study [22]. Fifty-one patients (average age: 66.3 ± 12.54) with colorectal carcinoma (stage I: 10, stage II: 19, stage III: 16, and stage IV: 6 cases) and 5 control (average age: 62.5 ± 11.08) were included the study. TNM and American Joint Committee on Cancer classifications were used for the stage of cancer.

### 2.2. RNA extraction and real-time PCR analyses

EZ-RNA Total RNA extraction kit (BI, Israel, Cat. No: 20-400-100) was used for RNA extractions of tissues and peripheral blood samples. Nanodrop ND-1000 spectrophotometer V3.7. was used for determining RNA amount and RNA purity. cDNA was obtained from 1 μg of total RNA by using iScript Reverse Transcription Supermix (Biorad, USA, Cat. No:170884). *CDH1*, *FHIT*, *PTEN*, and *TTPAL* genes expression levels were analysed by Rotor Gene-Q (Qiagen, Hilden, Germany). The reaction mix was prepared with iTaq Universal SYBR Green Supermix (Biorad, USA, Cat. No: 1725122) and oligonucleotide primers (designed by Genometry Biotechnology; *CDH1*, *TTPAL*, *FHIT*, *TBP*; İzmir, TURKEY and designed by Oligomere Biotechnology; *PTEN*, *GAPDH*; Ankara, TURKEY). Primers were designed based on following sequences as shown in Table.

We used the following real-time PCR protocol for *PTEN, CDH1, GAPDH*: 95 °C for 30 s initial denaturation followed by 40 cycles of 95 °C for 5 s and 60°C for 30 s; and for *FHIT*, *TTPAL* and *TBP:* 95 °C for 180 s initial denaturation followed by 35 cycles of 95 °C for 5 s, 58 °C for 10 s and 72 °C for 20 s.

### 2.3. Statistical analysis

There are some freely available software packages that support statistical analysis of expression results. REST 2009 V2.0.13 and SPSS v.19 Software [23] were used for assessing the relative expression results.

## 3. Results

### 3.1. mRNA levels of *CDH1*, *FHIT*, *PTEN*, and *TTPAL* genes

**Figure 1:**
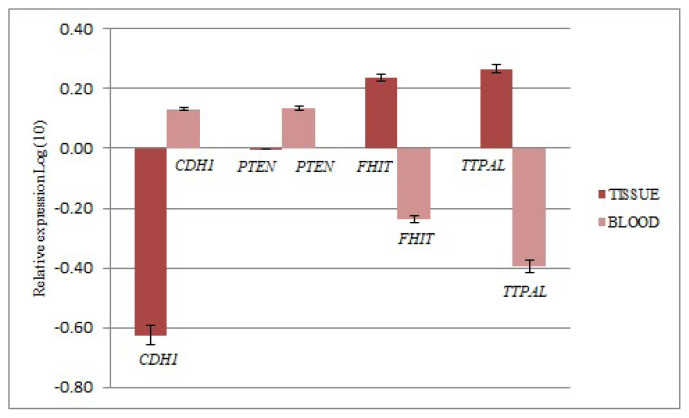
The results of real-time PCR analysis. The up/down-regulation of genes in tissues and blood of colorectal cancer (CRC) patient were given as fold regulation levels transformed to log values. *GAPDH* and *TBP* were reference genes for normalization.

Alteration of mRNA levels of *CDH1*, *FHIT*, *PTEN*, and *TTPAL* genes in tumor tissues was determined compared to control tissues. *GAPDH* (Glyceraldehyde-3-Phosphate Dehydrogenase) and *TBP* (TATA-Box Binding Protein) genes were used for the normalization. While the mRNA levels of *CDH1* decreased compared to the control group (0.247, fold regulation value: FRV) the mRNA levels of the *FHIT* and *TTPAL* genes increased (1.722 and 1.847 FRV, respectively). There were no *PTEN* gene expression differences at all tumor tissues compared to the control (0.997; FRV) [The final gene expression results were transformed to log values (any log base). This would make data distribution symmetric] (Figure 1).

Alteration of mRNA levels of *CDH1*, *FHIT*, *PTEN*, and *TTPAL* genes in peripheral blood of CRC cases were determined compared to the control blood. The mRNA levels of the *CDH1* and *PTEN* genes were increased (1.355 and 1.366, FRV; respectively), while the mRNA levels of *FHIT* and *TTPAL* genes decreased (0.580 and 0.403, FRV, respectively) (Figure 1).

### 3.2. mRNA levels of *CDH1*, *FHIT*, *PTEN*, and *TTPAL* genes at stage I-II-III-IV in tumors

**Figure 2:**
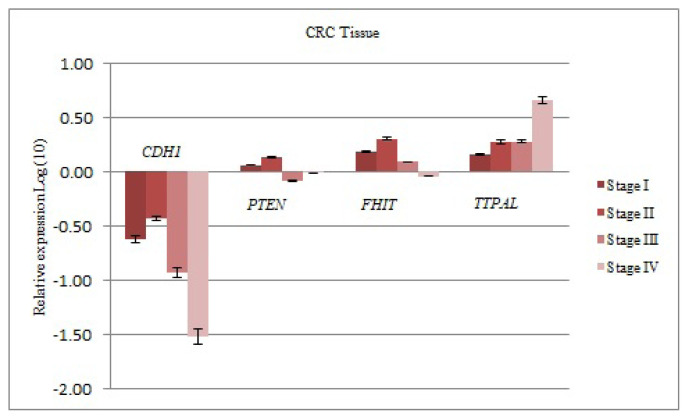
The results of real-time PCR analysis. The up/down-regulation of genes in tissues of CRC patients were given as fold regulation levels transformed to log values. *GAPDH* and *TBP* were reference genes for normalization.

The mRNA levels of the *CDH1* gene decreased at all stages (I–IV) compared to the control group [0.235; 0.369; 0.117; 0.030, FRV; respectively]. This downregulation was significant at stage III (P < 0.001). The mRNA levels of the *PTEN* gene increased at stage I–II (1.143; 1.331, FRV; respectively), decreased at stage III–IV (0.810; 0.962, FRV; respectively). The mRNA levels of the *FHIT* gene increased at stage I-II-III (1.505; 2.006; 1.218, FRV; respectively), decreased at stage IV (0.900, FRV). The mRNA levels of the *TTPAL* gene increased at all stages (I–IV) (1.417; 1.866; 1.875; 4.567, FRV; respectively) (Figure 2).

### 3.3. mRNA levels of *CDH1*, *FHIT*, *PTEN*, and *TTPAL* genes at stage I-II-III-IV in blood

**Figure 3:**
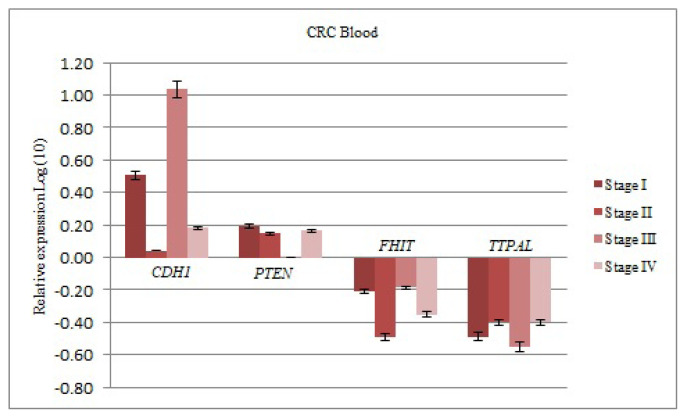
The results of real-time PCR analysis. The up/down-regulation of genes in blood of CRC patients were given as fold regulation levels transformed to log values. *GAPDH* and *TBP* were reference genes for normalization.

The mRNA levels of *CDH1* gene increased at all stages (I–IV) compared to the control group [3.212; 1.103; 10.853; 1.523, FRV; respectively]. This upregulation was significant at stage III (P < 0.05). The mRNA levels of the *PTEN* gene also increased at stage I-II-IV compared to the control group (1.557; 1.408; 1.455, FRV; respectively). There were no expression differences at stage III compared to the control (1.006; FRV). The mRNA levels of *FHIT* gene decreased at all stages (I–IV) compared to the control group (0.618; 0.323; 0.650; 0.445, FRV; respectively). The mRNA levels of the *TTPAL* gene also decreased at all stages (I–IV) compared to the control group (0.325; 0.398; 0.282; 0.398, FRV; respectively) (Figure 3).

## 4. Discussion

The presented study analysed the expression of *CDH1*, *PTEN*, *FHIT*, and *TTPAL* genes using tissue samples and blood of patients with different stages of colorectal cancer (CRC). Based on the information obtained from thousands of genetic variants discovered, cancer has been shown to be associated with other diseases and complex features. It has been clarified that single nucleotide polymorphisms and somatic copy number changes associated with the disease frequently affect gene expression levels [24–27].

Genetic susceptibility to colorectal and gastric carcinoma has been correlated with *CDH1* (E-cadherin) mutations [28–30]. Besides, infiltrative tumor growth pattern and lymph node metastasis were associated with loss of *CDH1* expression in CRC [31]. Downregulation or loss of E-cadherin has been suggested as a biomarker for colorectal cancer [32]. Similarly, in our study *CDH1* gene expression downregulated in tumor tissues at all stages of the disease compared to control tissues. On the contrary to this result, *CDH1* gene expression was upregulated in blood samples at all stages of the disease compared to control bloods. Methylation of EMT associated genes is related to the progression and prognosis of CRC [33]. Zheng et al. [34] suggested that intron mutation, gene methylation, and single nucleotide polymorphism may also affect *CDH1* expression. The discrepancy may result from these factors reported by Zheng et al. [34].

Yazdani et al. [35] reported that *PTEN* expression is important in CRC development. They found that negative *PTEN* expression was statically associated with tumor size and advanced TNM stages in patients with colorectal carcinoma. Loss of *PTEN* expression was reported in adenomas, adenomatous polyps, and CRC in some studies [36–38]. Sun et al. [39] suggested that PTEN might be a useful marker for the early diagnosis of CRC. They thought that, if *PTEN* was downregulated in adenomas, *PTEN* has to be involved in an early event during transformation. However, Molinari and Frattini [40] suggested that “there are conflicting results and, therefore it has not been clarified whether *PTEN* might play a prognostic role in CRC”. Although *PTEN* expression level tended to decrease in CRC, a decrease of *PTEN* gene expression in tumor tissue was not observed in our study.

*FHIT* gene expression in colon cancer was investigated by various methods in colon cancer [41–44]. However, studies related *FHIT* gene for colon cancer tumorigenesis are limited. Wierzbicki et al. [43] found high *FHIT* gene expression in adenomas and CRC. In their study, immunohistochemical analyzes showed comparable results. Thiagalingam et al. [45] reported a very high mRNA level of *FHIT* gene coding transcript in cell lines derived from human colorectal cancers. These findings are consistent with our study. The mRNA levels of the *FHIT* gene increased at stage I-II-III. Upregulation of the *FHIT* gene reported in tumor tissues was different from the blood samples. On the contrary to these findings, Kapitanovic et al. [46] reported that “expression of *FHIT* mRNA was significantly decreased in colon tumors relative to that in corresponding normal tissue”. However, the mRNA levels of *FHIT* gene decreased at all stages (I–IV) in blood compared to the control group in our study.

In the present study, the mRNA levels of the *TTPAL* gene increased in tissues at all stages (I–IV) and decreased in blood at all stages (I–IV). Similar to our findings, Gou et al. [21] reported that *TTPAL* expression at both mRNA and protein levels was detected in some colon cancer cells, but not in normal colon tissues. Besides, they reported that *TTPAL* mRNA expression was significantly upregulated in primary colorectal cancer tumors as compared with their adjacent normal tissues. Besides, Liu et al. [47] reported that the expression of *TTPAL* was significantly upregulated in gastric cancer compared to nontumor tissues. Also, suggested that DNA copy number was positively correlated with *TTPAL* expression because in the DNA copy number amplification group *TTPAL* expression level was higher compared to no amplification group. “Analysis of copy number of *TTPAL* in cancers from TCGA studies demonstrated that *TTPAL* was preferentially and more frequently amplified in colorectal cancers as compared with other cancer types, suggesting its particular involvement in colorectal cancer” [21].

We analysed the expression of mRNAs for *CDH1*, *PTEN*, *FHIT*, and *TTPAL* by real-time PCR and observed downregulation of *CDH1* and upregulation of *FHIT* and *TTPAL* in the tumor tissues. The expression levels of *CDH1, FHIT*, and *TTPAL* genes expression situations at tumoral tissues compared to control in line with the literature. This study also reports that there was an increase in *TTPAL* expression as the stage progresses in tumor tissues of patients, but no significant difference was observed. Although related expression levels in tissue did not correlate with its expression in blood, consistent with previous studies *FHIT* and *TTPAL* genes upregulation and *CDH1* downregulation, in especially tumoral tissues, may serve as predictive determinants for the patients with colorectal cancer. However, the clinical application of these genes as a biomarker remains unclear for colorectal cancer. Besides, the discordant results may result from the size of the analysed study group or the methods of determining gene expression levels. In conclusion, replications studies in much larger study groups are required before suggesting that these genes are predictive markers.

## Figures and Tables

**Table:** Primer sequences of the analysed genes.

Gene symbol	Primer sequences
*CDH1-F*	5’-CCCTTCCTCAAAACACACTCC-3’
*CDH1-R*	5’-TGGCAGTGTCTCTCCAAATC-3’
*FHIT-F*	5’-GGACTTTCCTGCCTCTTGGAGA-3’
*FHIT-R*	5’-GCGGTCTTCAAACTGGTTGCCA-3’
*PTEN-F*	5’-TGGATTCGACTTAGACTTGACCT-3’
*PTEN-R*	5’-GGTGGGTTATGGTCTTCAAAAGG-3’
*TTPAL-F*	5’-CCACTCCATCTCCTCAATCAACC-3’
*TTPAL-R*	5’-CTCCACACACTTCACTCACACC-3’
*TBP-F*	5’-TCTATCCACACTCAATCTTCCTTC-3’
*TBP-R*	5’-CCTTCCTCCCTCTCTTATCCTC-3’
*GAPDH-F*	5’-CATTGCCCTCAACGACCACTTT-3’
*GAPDH-R*	5’-GGTGGTCCAGGGGTCTTACTCC-3’.

## Footnotes

1 Cancer Index [online]. Website: http://www.cancerindex.org [accessed 05 October 2021].
